# Near-infrared quantum dots labelled with a tumor selective tetrabranched peptide for in vivo imaging

**DOI:** 10.1186/s12951-018-0346-1

**Published:** 2018-03-03

**Authors:** Jlenia Brunetti, Giulia Riolo, Mariangela Gentile, Andrea Bernini, Eugenio Paccagnini, Chiara Falciani, Luisa Lozzi, Silvia Scali, Lorenzo Depau, Alessandro Pini, Pietro Lupetti, Luisa Bracci

**Affiliations:** 1 Department of Medical Biotechnologies, University of Siena, 53100 Siena, Italy; 2 Department of Life Sciences, University of Siena, 53100 Siena, Italy; 3 Department of Biotechnology, Chemistry and Pharmacy, University of Siena, 53100 Siena, Italy

**Keywords:** Peptides, Near-infrared quantum dots, In vivo imaging

## Abstract

**Background:**

Near-infrared quantum dots (NIR QDs) are a new class of fluorescent labels with excellent bioimaging features, such as high fluorescence intensity, good fluorescence stability, sufficient electron density, and strong tissue-penetrating ability. For all such features, NIR QDs have great potential for early cancer diagnosis, in vivo tumor imaging and high resolution electron microscopy studies on cancer cells.

**Results:**

In the present study we constructed NIR QDs functionalized with the NT4 cancer-selective tetrabranched peptides (NT4-QDs). We observed specific uptake of NT4-QDs in human cancer cells in in vitro experiments and a much higher selective accumulation and retention of targeted QDs at the tumor site, compared to not targeted QDs, in a colon cancer mouse model.

**Conclusions:**

NIR QDs labelled with the tetrabranched NT4 peptide have very promising performance for selective addressing of tumor cells in vitro and in vivo, proving rising features of NT4-QDs as theranostics.

**Electronic supplementary material:**

The online version of this article (10.1186/s12951-018-0346-1) contains supplementary material, which is available to authorized users.

## Background

Quantum dots (QDs) with unique optical properties are a class of novel nanomaterials and have been extensively used in medical research [1, 2]. QDs are nanocrystals with diameters ranging from 2 to 10 nm and when coated with water-soluble bioactive material, they form core–shell nanostructures that are water soluble and biocompatible.

QDs can be conjugated with different molecules, such as antibodies or peptides, to obtain target-selective functional fluorescent nanodevices with unique optical properties. These functional QD probes have been increasingly used for cell and molecular tracing, in in vivo tumor imaging and drug delivery [3, 4].

Near-infrared QDs (NIR QDs) with emission wavelengths between 700 and 900 nm have unique optical properties, such as high fluorescent intensity and sensitivity, high spatial resolution, excellent fluorescent stability, deep tissue penetration, minimum photo-damage to biological samples and minimum interference from background auto-fluorescence by biomolecules in living systems [5–8]. In addition QDs have an electron density compatible with imaging by transmission electron microscopy. Therefore NIR QDs have great potential for early diagnosis of cancer, in vivo tumor imaging, ex vivo ultrastructural studies on tumor cells, and personalized tumor therapy [9–11].

In this paper, we report in vitro and in vivo tumor targeting and imaging by NIR QDs functionalized with tetra-branched NT4 peptides, which are very promising cancer theranostics by virtue of their already established high cancer selectivity.

In previous papers, we demonstrated that NT4 very selectively binds to human cancer tissues in different malignancies and can efficiently and selectively deliver drugs or liposomes for cancer cell imaging or therapy, in vitro and in vivo [12–17]. We demonstrated that NT4 specifically binds to sulfated glycosaminoglycans and LRP receptors on cancer cells and tissues. Furthermore, NT4 can interfere with cancer cell migration and adhesion and may therefore reduce tumor aggressiveness and metastatic potential [18, 19]. We reported that conjugation of paclitaxel with NT4 leads to increased therapeutic activity of the drug in an orthotopic model of breast cancer in mice and produces tumor regression which is not achieved with unconjugated paclitaxel under identical experimental conditions [20].

## Results

### Conjugation of NIR QDs with NT4

**Scheme 1:**
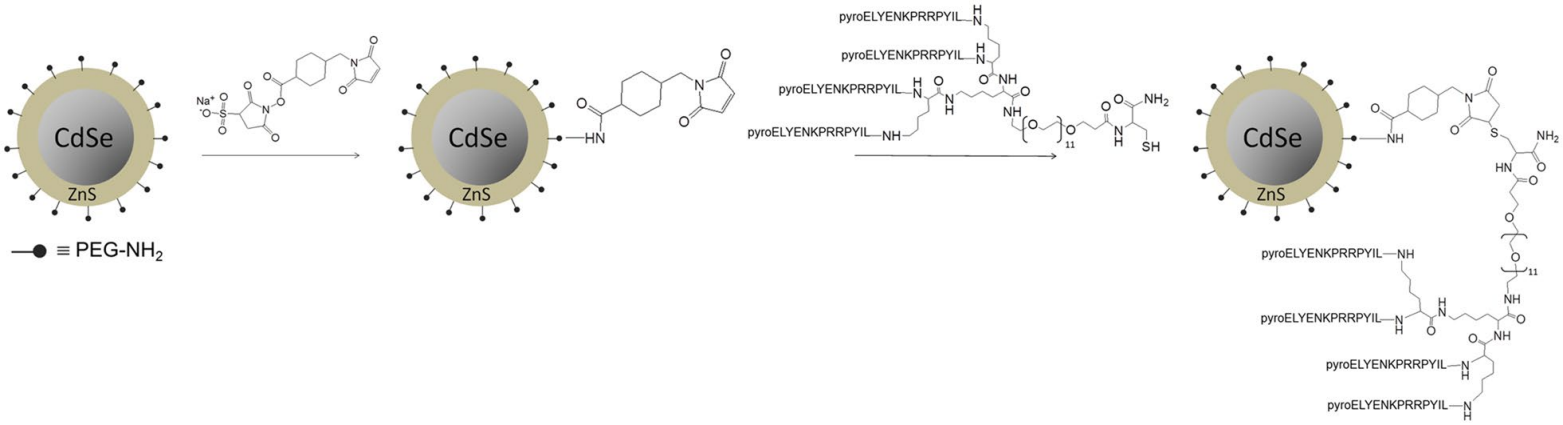
Synthesis of NT4-QDs

NT4 peptide was conjugated with NIR QDs using commercially available QDs functionalized with amine-derivatized PEG (Alexa Fluor 705 nm quantum dots, Invitrogen). The amine was first conjugated with a bifunctional cross-linker, sulfo-SMCC. Then NT4 was added to the maleimide double bond via the thiol group of the C-terminus cysteine (Scheme 1).

NT4-QDs were purified using superdex 200 by size exclusion chromatography. The concentration of purified NT4-QDs in the eluate was calculated to be 250 nM on the basis of the extinction coefficient at 532 nm, namely 2.1 × 10^6^/(mol/l)/cm, according to the supplier’s instructions.

### NMR characterization

**Fig. 1:**
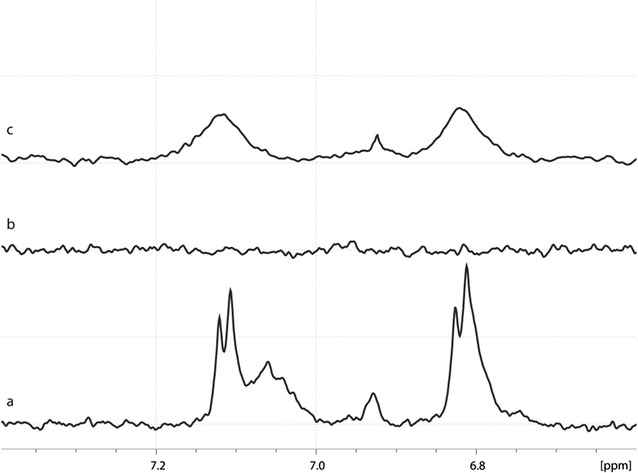
NMR. Region of tyrosine aromatic protons from NMR spectra of (a) free NT4, (b) free QDs and (c) NT4-QDs. Note the absence (b) and presence of broadened peaks (c) compared to (a)

To characterize NT4-QDs, NMR spectra of free NT4, free QDs and QD-bound NT4 were acquired and compared. In particular, resonances of tyrosine δ- and ε-protons were considered because (i) they are the only aromatic residues in the NT4/QD system and give sharp, intense peaks easy to assign and to follow, (ii) the peak intensities of the eight tyrosines from NT4 added up, enhancing resolution even at very low concentration and were insensitive to pH variations. The free NT4 one-dimensional proton spectrum showed tyrosine aromatics centred at the typical random coil chemical shift of 6.95 ppm with little or no differentiation, diagnostic of an unstructured peptide (see Fig. 1a). The QD sample spectrum showed no resonances at all in the same spectral range (see Fig. 1b), while NT4-QDs showed tyrosine peaks, indicating successful binding of the peptide to the nanoparticles (see Fig. 1c). Moreover, the aromatic signals underwent considerable broadening, diagnostic of increased correlation time due to slow tumbling of the linked nanoparticle. The chemical shift did not change, suggesting that no conformational effect was exerted by QDs on the peptide. By calibration with the internal TSP standard, we estimated an approximate NT4/QD ratio of 8:1 (see Additional file 1: Figure S1 for details and full NMR spectra).

### In-vitro characterization of NT4-QDs

**Fig. 2:**
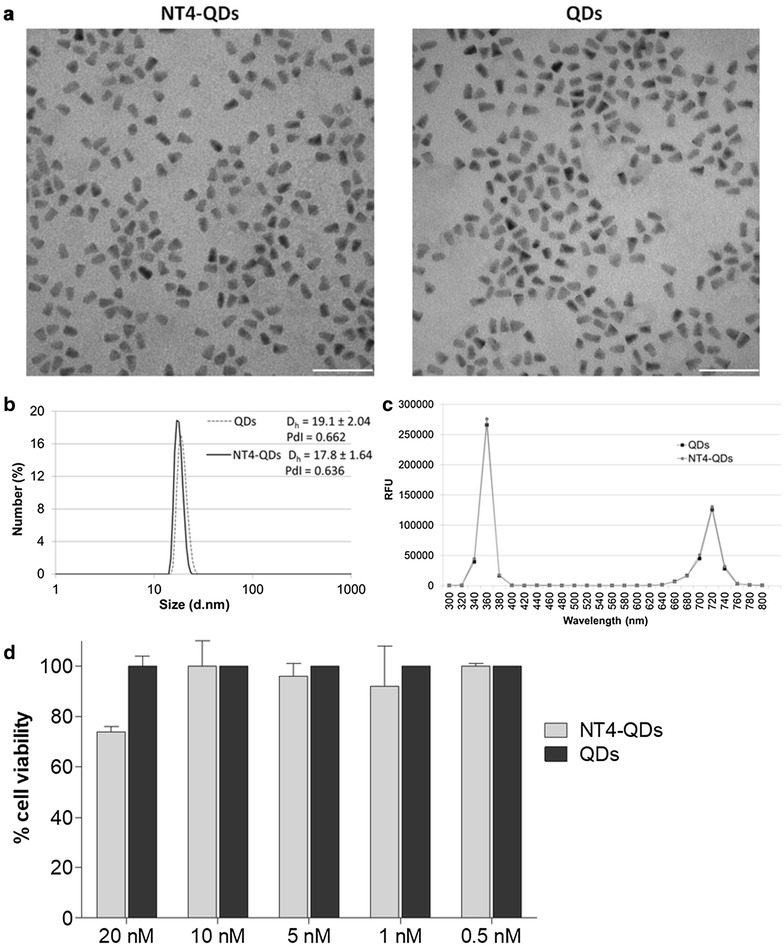
Characterization of NT4-QDs. **a** TEM image of monodispersed NT4-QDs and unlabelled QDs. Scale bar 50 nm. **b** Dynamic light scattering of NT4-QDs (black line) and QDs (dotted grey line) in PBS buffer after 24 h at 25 °C. The hydrodynamic diameter (D_h_) ± SD and polydispersity index (PdI) are reported in the figure. **c** Fluorescence spectrum of the same concentration (240 nM) of NT4-QDs and unlabelled QDs. **d** Cytotoxicity of NT4-QDs and free QDs in HT29 human colon adenocarcinoma cell line after 24 h incubation at 37 °C

NT4-QDs were analysed by transmission electron microscopy (TEM) and proved to be well dispersed as single particles (Fig. 2a). The hydrodynamic diameter of NT4-QDs and QDs was determined by dynamic light scattering (DLS) in PBS pH 7.4 and in water at different time intervals (time 0 and 24 h). The average size of NT4-QDs and QDs when tested at 24 h incubation in PBS, was 17.8 and 19.1 nm, respectively (Fig. 2b and Additional file 1: Figure S2). The hydrodynamic diameter did not change over time and in different buffers (not shown). The fluorescence properties of modified QDs were also assessed at 300–800 nm. The fluorescence spectrum of equal concentrations of NT4-QDs and QDs showed identical peaks of absorption and emission, proving that the modification of the QD surface did not impact on the fluorescence properties of the final product (Fig. 2c).

Cytotoxicity of NT4-QDs, compared to that of unconjugated QDs, was tested in vitro in HT-29 human adenocarcinoma cell line (Fig. 2d). NT4-QDs did not show any significant cytotoxic activity. At 20 nM, which was the highest concentration of NT4-QDs, slight cytotoxic activity was observed after 24 h incubation.

### In vitro cell binding of NT4-QDs

**Fig. 3:**
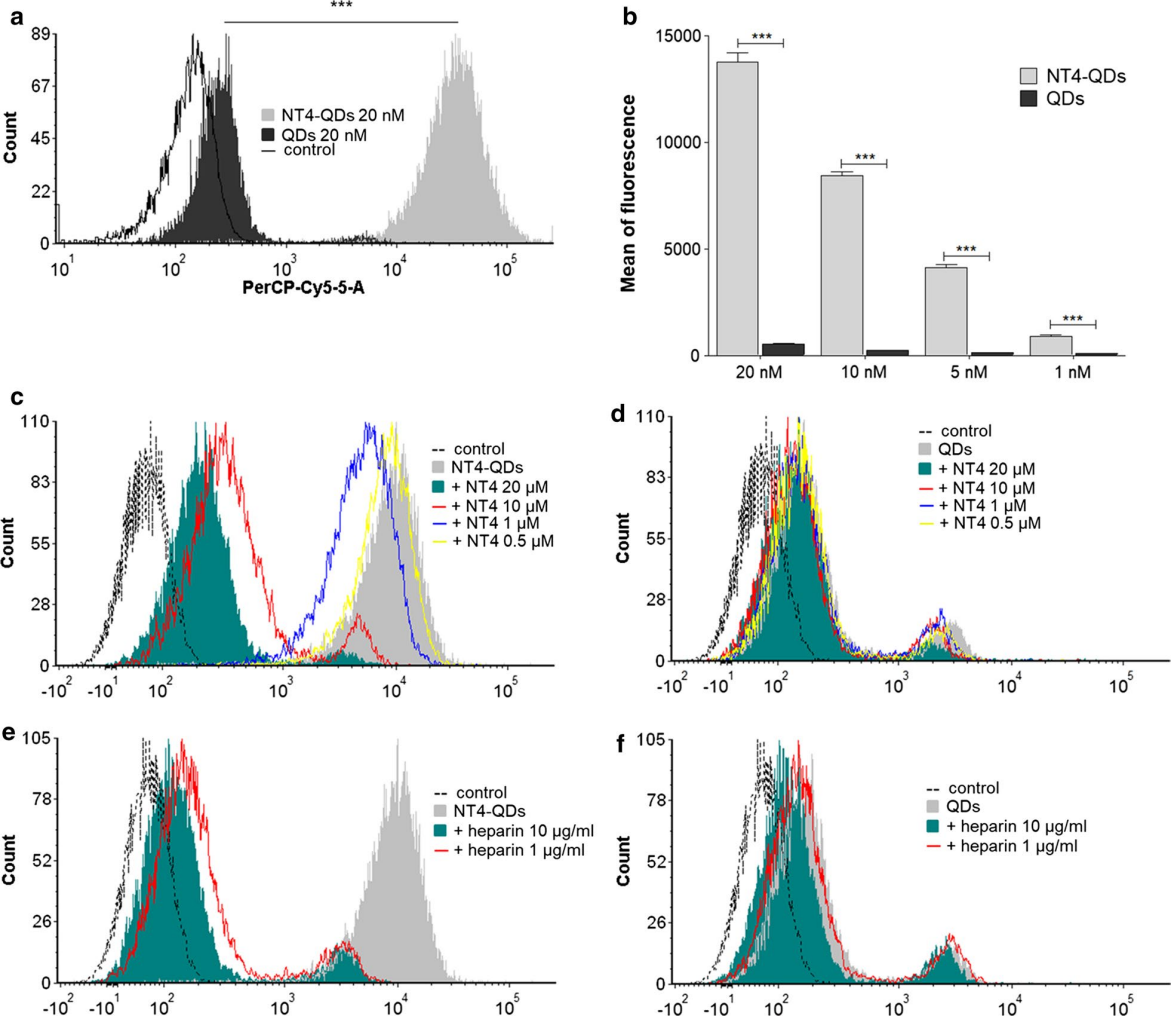
NT4-QDs binding by flow cytometry. **a** Flow cytometry analysis showing binding of 20 nM NT4-QDs (light grey) and unlabelled QDs (dark grey) to HT29 cells. **b** Mean fluorescent intensity using different concentrations of NT4-QDs and unlabelled QDs. ***p < 0.001 calculated using one tailed Student *t* test. NT4-QDs (**c**) and unlabelled QDs (**d**) binding in the presence of NT4. NT4-QDs (**e**) and unlabelled QDs (**f**) binding in the presence of heparin. Flow cytometric analysis on 10,000 events was done using a BD FACSCanto II instrument (BD, NJ. USA) using a blue laser dye and the PerCP-Cy5-5-A channel

Binding of NT4-QDs was tested in HT29 human colon adenocarcinoma cell line by flow cytometry (Fig. 3). Cells, incubated with 20 nM NT4-QDs showed a fluorescence signal two log higher than unlabelled QDs and the difference proved statistically significant (Fig. 3a; p < 0.001). Binding of scalar concentrations of NT4-QDs to HT29 cells produced a dose-dependent fluorescent signal with very good statistical significance (Fig. 3b). Binding of NT4-QDs was inhibited by either unlabelled NT4 or heparin, while the much lower signal of unlabelled QDs was not affected, which confirms the specificity of NT4-QDs cell binding (Fig. 3c–f).

**Fig. 4:**
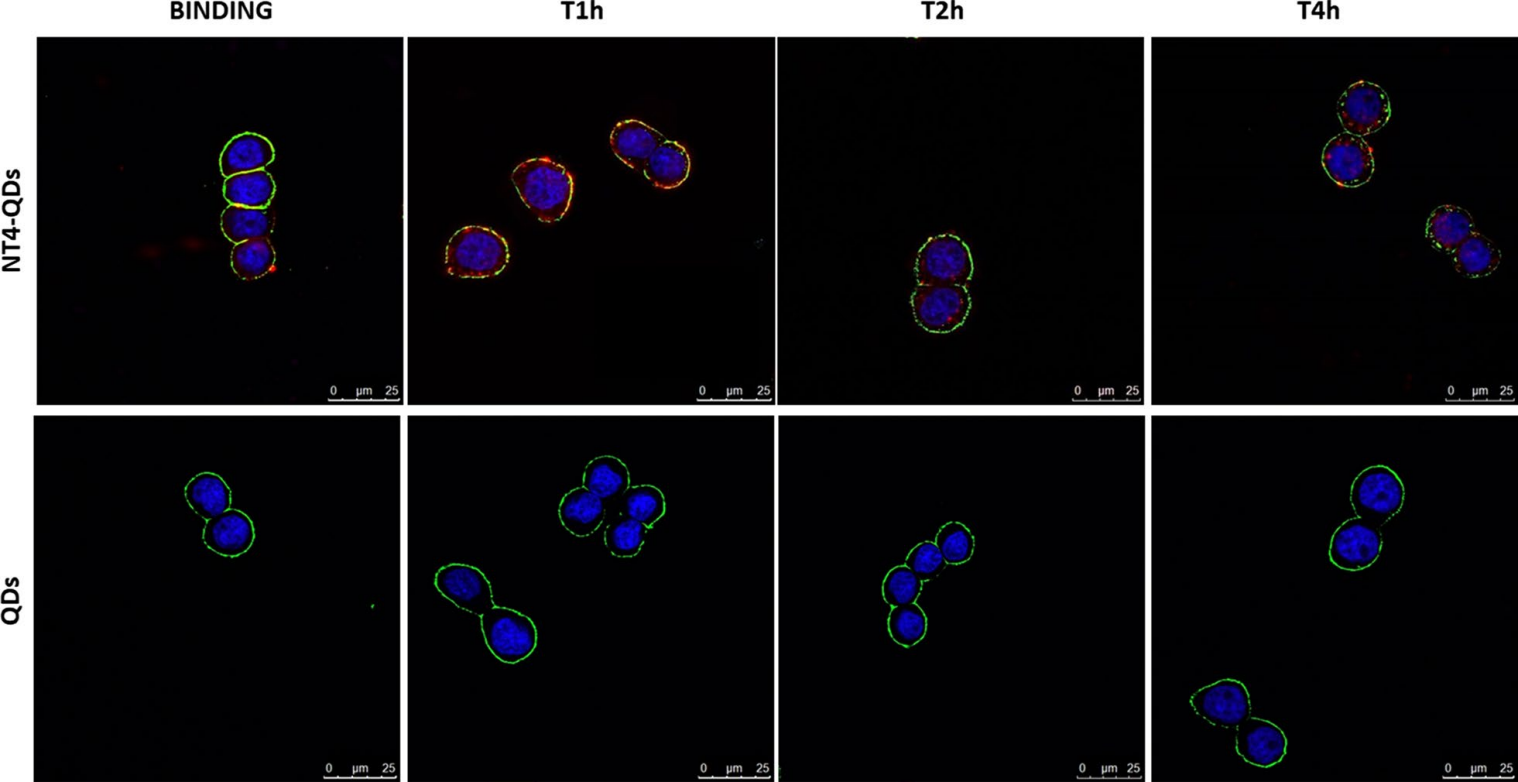
Binding and internalization (T 1, 2, and 4 h) of NT4 conjugated with NIR QDs (red) on PANC-1 human pancreas adenocarcinoma cells. Nuclei are stained with DAPI (blue) and plasma membranes are stained with wheat germ agglutinin Alexa Fluor 488 (green)

Cell binding and internalization of NT4-QDs were analysed in HT29 by immunofluorescence (Fig. 4). At time 0 (binding, detected after 30 min of incubation), NT4-QDs (20 nM, red signal) were localized on cell membranes. At the following incubation times, NT4-QDs were clearly localized intracellularly. No signal was detected with unlabelled QDs.

**Fig. 5:**
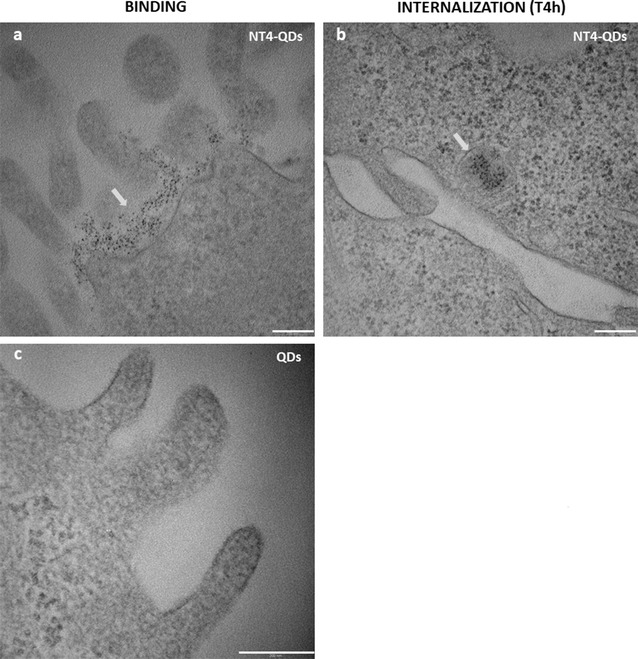
Transmission electron micrographs of HT-29 cell line incubated with NT4-QDs (**a**, **b**) or with unlabelled QDs (**c**). NT4-QD clusters: **a** localized at cell membrane after 30 min of incubation (binding), **b** entrapped in vesicles inside cells after 4 h of incubation. Scale bar 200 nm

The trafficking of NT4-conjugated particles inside the cells was also monitored by TEM (Fig. 5). Particles entered the cells by an endocytic-like pathway. A cluster of particles localized on the cell membrane at 30 min (Fig. 5a) was then engulfed by vesicles and internalized at 4 h (Fig. 5b).

### In vivo imaging of NT4-QDs

**Fig. 6:**
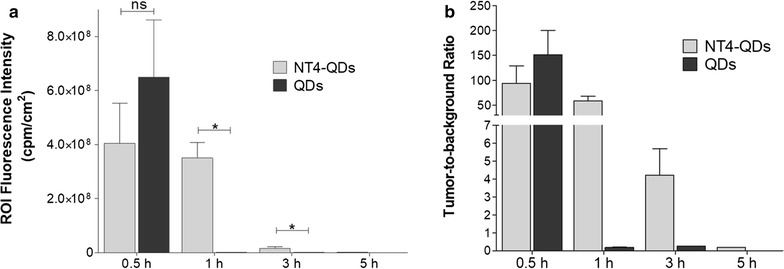
In vivo NIR fluorescence imaging of HT29 tumor-bearing mice injected with 200 pmol of NT4-QDs (n = 3) and nude QDs (n = 3). ROI Fluorescence intensity (**a**) and tumor-to-background ratio (**b**) measured at different time intervals in mice injected with NT4-QDs (light grey) or QDs (dark grey). The data is represented as mean ± SD. *p < 0.05 compared to mice injected with unconjugated QDs (two-tailed Student’s *t* test and GraphPad Prism 5)

Athymic nude mice bearing HT29 xenograft tumors (2 weeks post inoculation of 1 × 10^6^ cells, tumor size about 0.6–0.8 cm^3^) were injected with NT4-QDs or unlabelled QDs (200 pmol of QDs per animal) in the tail vein. The mice were imaged at many time points post-injection using the Calliper in vivo imaging system (Fig. 6 and Additional file 1: Figure S3).

The excitation filter was set at 585 nm and the emission filter at 660 nm to take fluorescence images with a strong fluorescent signal and low background signal.

Monitoring of tumor fluorescence intensity showed that as early as 0.5 h post-injection, the fluorescent signal of NT4-QDs and unlabelled QDs appeared in the tumors with higher fluorescent intensity of the latter with respect to the former (Fig. 6a).

After 1 h, we detected a higher NIR fluorescent signal at the tumor site in mice injected with NT4-QDs than in those injected with QDs. At 1 h, nude QDs only showed background fluorescence, and the ratio of fluorescent NT4-QDs:QDs was 318:1 (Fig. 6b), indicating that tumor targeting by NT4-QDs induced much higher retention of QDs at the tumor site than targeting by nude QDs.

## Discussion

Optical imaging in vivo provides real-time tumor visualization, thus not only allowing diagnosis but also possible applications in intraoperative image-guided surgery. Fluorescent probes in the near infrared (NIR) have a much larger range of in vivo applications than conventional fluorophores because they overcome the problem of tissue autofluorescence.

Quantum dots in the NIR are of great interest for in vivo biological imaging and diagnostics. Compared to traditional organic dyes, QDs have the advantage of wavelength-tunable emission, broad excitation spectra, sharp fluorescent peak, and high photostability. The major problem limiting the use of QDs in human clinical applications is their inherent toxicity, which is influenced by many factors and depends on different types of functionalized QDs, their core and shell structures, size and surface charge [21, 22].

An important way to minimize QD toxicity is surface coating and modification, for example to improve water solubility, stability and biocompatibility, or to assign a desired bioactivity. For example, coating QDs with hydrophilic polyethylene glycol (PEG) groups can increase stability and reduce aggregation. Coupling QDs with targeting moieties allows more selective tumor uptake and retention, thus facilitating detection of tumors and cancer cells in vivo and reducing systemic toxicity.

In previous studies we showed that a tetra-branched form of neurotensin, called NT4, has extraordinary selectivity towards different human tumors (i.e. colon, pancreas and urinary bladder cancer) and is easily coupled to chemical entities or liposomes for cancer cell killing or imaging [12–17]. In the present study, the tetrabranched NT4 peptide was conjugated with NIR QDs functionalized with amine-derivatized PEG and analysed for in vitro and in vivo tumor targeting and imaging. NT4-QDs were characterized by DLS, NMR analysis, transmission electron microscopy and in vitro cytotoxicity assays, revealing that NT4 conjugation with NIR-QDs did not change nanoparticle morphology or aggregation state and did not significantly affect cell viability. The selective internalization of NT4-QDs and nude QDs in tumor cells was analysed by confocal microscopy and transmission electron microscopy. In vivo experiments in HT29 xenografted mice showed much higher retention of NT4-conjugated than unconjugated QDs at the tumor site.

The results achieved so far indicate that NT4-labelled NIR QDs have very promising performance for selective addressing of tumor cells.

## Methods

### Peptide synthesis

Solid-phase synthesis was carried out on a MultiSynTech Syro automated multiple peptide synthesizer (Witten, Germany), employing Fmoc chemistry with 2-(1*H*-benzotriazole-1-yl)-1,1,3,3-tetramethyluronium hexafluorophosphate/*N*,*N*-diisopropylethylamine (HBTU) activation. NT4-Cys was synthesized on Novasyn TGR resin using Fmoc-Cys(Trt)-OH as first, and Fmoc-PEG12-OH (Iris Biotech, Germany) as second coupling step. Then two coupling steps with Fmoc-Lys(Fmoc)-OH were used to build the core. Pyro-Glu-OPentachlorophenylester was used for the N-terminal acid of the neurotensin sequence. Peptides were cleaved from the resins and deprotected by treatment with trifluoroacetic acid containing water and triisopropylsilane (95:2.5:2.5) for 1.5 h at room temperature. After precipitation with diethyl ether, branched peptides were purified by RP-HPLC. Final peptide purity was confirmed to be over 99% by HPLC on a C18 Jupiter column (Phenomenex, 300 Å, 5 μm, 250 × 4.6 mm) using 0.1% TFA/water as eluent A and methanol as eluent B with a linear gradient from 80% A to 5% A in 30 min. All peptides were characterized by UltraflexIII MALDI TOF/TOF mass spectrometry (Bruker Daltonics, Bremen, Germany). Commercial reagents, catalysts and ligands were used without further purification from freshly opened containers, unless otherwise stated.

### NT4-QD construction

Qdot 705 ITK amino PEG, used in this study, was commercially available (Qdot 705 ITK™ Amino (PEG) Quantum Dots, Molecular Probes). Amine-modified QDs were first conjugated with a bifunctional cross-linker, sulfo-SMCC and then with NT4-Cys peptide in the molar ratio 1 QDs: 20 linker: 50 peptide. Briefly, QDs were exchanged into phosphate buffered saline (PBS, pH 7.4) by adding 300 μl PBS and concentrating using a PES 100 K MWCO protein concentrator (Thermo Scientific) spun at 3800*g* at 4 °C. QDs were then conjugated with sulfo-SMCC (sulfosuccinimidyl-4-(*N*-maleimidomethyl)cyclohexane-1-carboxylate; Thermo Scientific) for 1 h at room temperature on a rotator to generate a maleimide-activated surface on the QDs, and free sulfo-SMCC was then removed using a NAP-5 column (GE Healthcare). The maleimide-functionalized QDs were incubated with NT4-SH for 1 h at room temperature on the rotator. 10 mM 2-mercaptoethanol was added to the reaction and left for an additional 30 min to cap unreacted maleimides. NT4-conjugated QDs were further purified via a Superdex 200 column eluting with 20 mM phosphate buffer (pH = 7.4). The final concentration of NT4-QDs was calculated by detecting absorption at 532 nm using a spectrophotometer (Jenway, UK) and using the extinction coefficient 2.1 × 10^6^/(mol/l)/cm as provided by the manufacturer. As a control, the absorption of nude QDs at the same concentration was measured and the resulting difference in absorption at 532 nm between QDs and NT4-QDs was + 5% ± 0.6.

### NMR analysis

NMR samples of NT4, QDs and NT4/QDs were prepared in aqueous PBS pH 7.4 with 10% D2O and a final concentration of 1.25 µM for QDs and NT4/QDs and of 10 µM for NT4 (to account for multiple conjugation of peptides to QDs). TSP with a concentration of 500 µM was used as internal standard. All spectra were acquired on a Bruker DRX Avance spectrometer operating at 14.1 Tesla at a temperature of 298 K. One-dimensional spectra were recorded by accumulation of 1024 FIDs over 8192 points. The spectral width was set to 6000 Hz and repetition delay to 3 s, water suppression was achieved by excitation sculpting [23].

### Dynamic light scattering

The size distribution (hydrodynamic diameter; dh) of QDs was determined by DLS using a Zetasizer Nano ZS from Malvern Instruments. NT4-QDs and QDs were diluted in PBS or water at the concentration of 270 nM and the hydrodynamic diameter (dh) and the polydispersity index (PdI) were measured at time 0 and after 24 h at 25 °C. Measures were repeated 20 times.

### Fluorescence spectrum

NT4-QDs and QDs were dissolved in PBS at 240 nM and the fluorescence spectrum was recorded in a SpectraMax M.

### Cell cultures

HT29 human colon adenocarcinoma was grown in McCoy’s 5a Medium supplemented with 10% fetal bovine serum, 200 µg/ml glutamine, 100 µg/ml streptomycin and 60 µg/ml penicillin and was maintained in 5% CO_2_. Cell lines were purchased from ATCC (The Global Bioresource Center).

### In vitro cytotoxicity assay

HT29 cells were plated at a density of 5 × 10^3^/well in 96-well microplates. Different concentrations, from 0.5 to 20 nM, of NT4-QDs and undecorated QDs were added 24 h after plating and cells were incubated for 1 days at 37 °C. Growth inhibition was assessed by 3-(4,5-dimethylthiazol-2-yl)-2,5-diphenyltetrazolium bromide (MTT). The experiment was performed twice in triplicate. EC50 values were calculated by non-linear regression analysis using Graph Pad Prism 5.03 software.

### NT4-QD binding by flow cytometry

100,000 cells/experiment were incubated in 96-well U-bottom plates for 30 min at room temperature with different concentrations of NT4-QDs (from 5 to 20 nM) in PBS-EDTA 5 mM-BSA 0.5%. Flow cytometric analysis on 10,000 events was done using a BD FACSCanto II instrument (BD, NJ. USA) using a blue laser dye and the PerCP-Cy5-5-A channel. Assays were performed in triplicate and the flow cytometry results were analysed by nonlinear regression analysis using GraphPad Prism 5.03 software.

### NT4-QD binding and internalization

The binding and internalization of NT4-QDs and free QDs were tested in HT29 cell line. 3 × 10^4^ cells/well were seeded on 24-well plates, grown for 24 h and then incubated with 20 nM of NT4-QDs or with unlabelled QDs for 30 min at 37 °C in PBS-1% BSA. After 30 min of incubation at room temperature, the cells were washed and grown in medium for 1, 2 or 4 h at 37 °C to allow peptide internalization.

### Immunofluorescence

Cells were fixed with PBS 4% formalin and then plasma membranes were stained with wheat germ agglutinin-Alexa Fluor 488 (2.5 µg/ml in PBS-1% BSA). Each step was followed by three washes in PBS. Samples, mounted using Prolong Gold antifade with DAPI (Molecular Probes) were analysed by confocal laser microscope (Leica TCS SP5) with 380 λex and 680–750 λem, 380 λex and 450–470 λem, and 488 λex and 510–550 λem for NIR QDs, DAPI and FITC, respectively. All images were processed using ImageJ software (NIH).

### Transmission electron microscopy

#### Analysis of QDs

5 µL of free QDs or NT4-QDs were loaded on carbon coated 300 mesh copper grid and after 30 s the excess was blotted with filter paper. Samples were analysed with a FEI Tecnai G2 SPIRIT transmission electron microscope operating at an electron accelerating voltage of 100 kV.

#### QDs internalization in HT29 cancer cells

Cells were fixed in 2.5% glutaraldehyde solution in phosphate buffer 0.1 M pH 7.2 (PB) for 2 h at 4 °C, washed in PB, post-fixed in 1% OsO_4_ in PB for 30 min at 4 °C, dehydrated in an ascending alcohol series, incubated twice in propylene oxide and finally infiltrated and embedded in epon/araldite resin that was polymerized at 60 °C for 48 h.

Ultrathin sections (60 nm) were cut from embedded samples on a Reichert-Jung Ultracut E ultramicrotome, mounted on 200-mesh copper grids, stained with uranyl acetate and lead citrate and observed in a FEI Technai G2 SPIRIT transmission electron microscope operating at an electron accelerating voltage of 100 kV.

### In vivo imaging

Athymic female nude mice (Charles River Laboratories, Inc.), 5 to 6 weeks of age, were injected s.c. in the flank with 1 × 10^6^ HT-29 cells. When tumors reached a diameter of 6–8 mm (2 weeks after tumor inoculation) mice were randomly divided into two groups and injected in the tail vein with 200 pmol of NT4-QDs and with undecorated QDs. Mice were imaged at many time points (0.5, 1, 3, 5 and 24 h) post-injection using the Calliper in vivo imaging system.

All experiments were conducted in accordance with the laws and regulations for experiments and procedures (Directive 2010/63/EU). All experimentation on live vertebrates described in this article was approved by the Italian Ministry of Health (Document No. 699/2015-PR).

## Additional file

**Additional file 1: Figure S1.** Full NMR spectra of free NT4 (A), free QDs (B) and NT4-QDs (C) (water region was deleted). **Figure S2.** DLS spectra of 270 nM NT4-QDs and QDs. **Figure S3.** In vivo imaging of HT29 tumor-xenograft mice (yellow circles) at 3 h post-injection of 200 pmol of NT4-QDs (left) or free QDs (right).

## Abbreviations

NIR: near-infrared
QDs: quantum dots
TEM: transmission electron microscopy
PEG: polyethylene glycol
