# Supplementary material for: Near-infrared quantum dots labelled with a tumor selective tetrabranched peptide for in vivo imaging
Source: J Nanobiotechnology. 2018 Mar 3;16:21. doi: 10.1186/s12951-018-0346-1 (PMC5834876; doi:10.1186/s12951-018-0346-1)
Supplement: Supplementary file 1 — Additional file 1: Figure S1. Full NMR spectra of free NT4 (A), free QDs (B) and NT4-QDs (C) (water region was deleted). Figure S2. DLS spectra of 270 nM NT4-QDs and QDs. Figure S3. In vivo imaging of HT29 tumor-xenograft mice (yellow circles) at 3 h post-injection of 200 pmol of NT4-QDs (left) or free QDs (right). [file 12951_2018_346_MOESM1_ESM.pdf]

Additional file

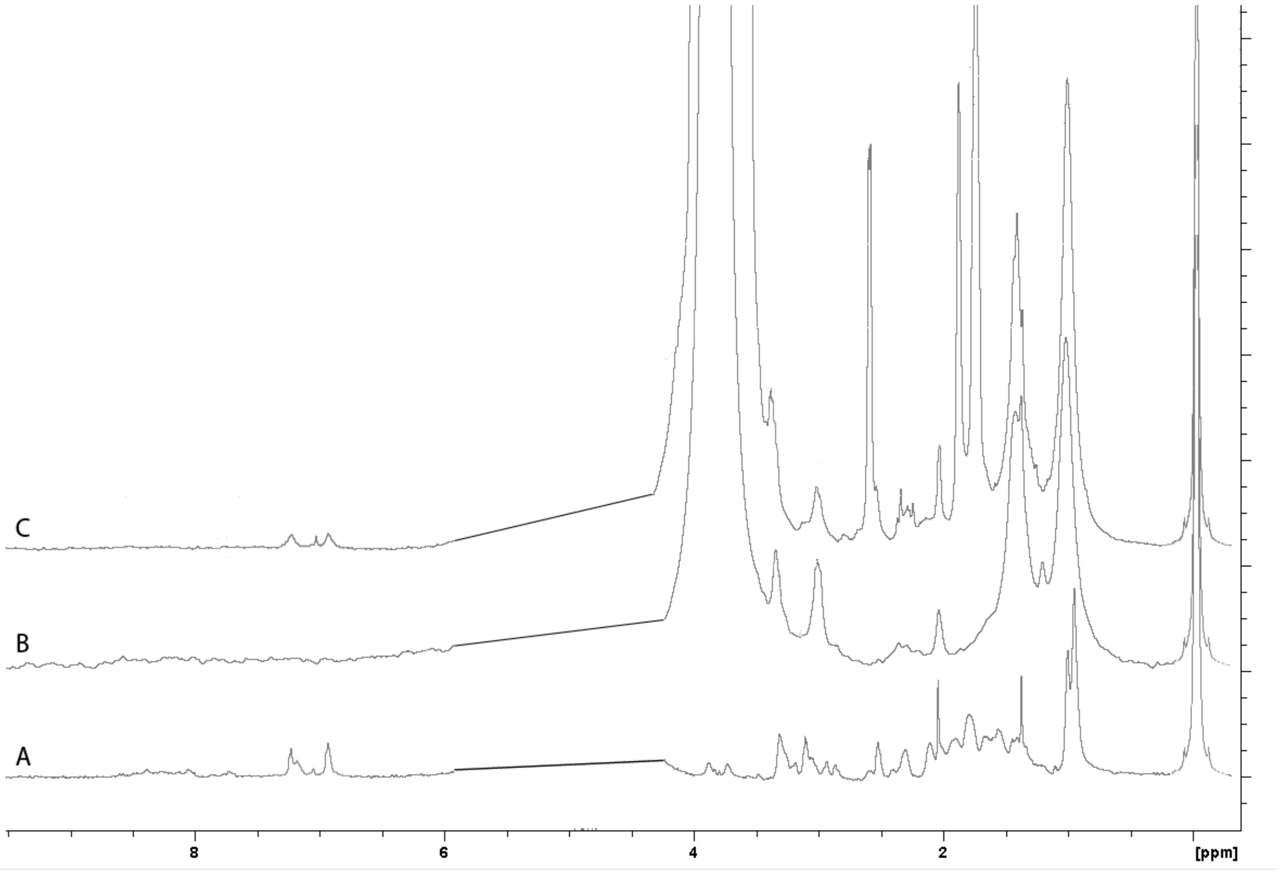

Figure S1. Full NMR spectra of free NT4 (A), free QDs (B) and NT4-QDs (C) (water region was deleted).

The relative concentration of a given molecule (i.e. NT4) was calculated in relation to the known concentration of the internal standard (i.e. TSP) using the areas of the corresponding resonances in the spectrum:

$$\frac{M}{S} = \frac{I_M}{I_S} \times \frac{N_S}{N_M}$$

where M and S are the concentrations of the molecule and the internal standard (S is known), I_M and I_S are the integrals of their signals and N_M and N_S are the number of protons contributing to these signals; for TSP: $N_S = 9$ and for NT4: $N_M = 32$ (NT4 carries 8 Tyrosines with 4 aromatic protons each).

The concentration of NT4 resulting from integrals of TSP (I_S) and aromatic protons of Tyrosines (I_M) was ~10 µM; by dividing such value for the concentration of QDs of 1.25 µM used in NMR experiments, an approximate NT4/QDs ratio of 8:1 on average was estimated.

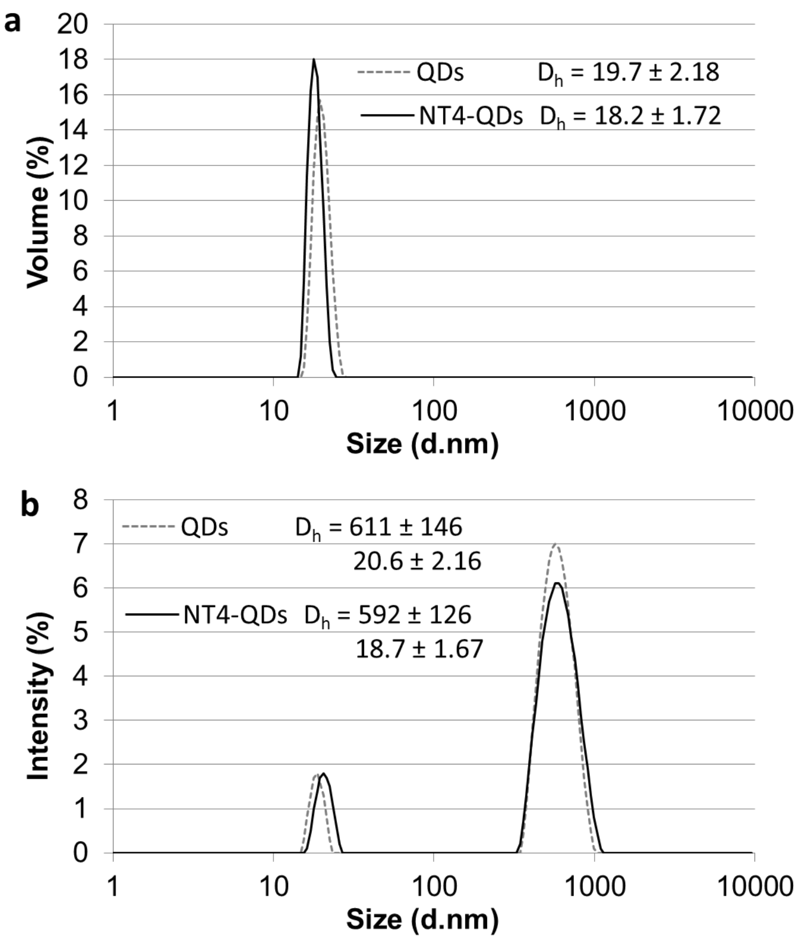

Figure S2. DLS spectra of 270 nM NT4-QDs and QDs.

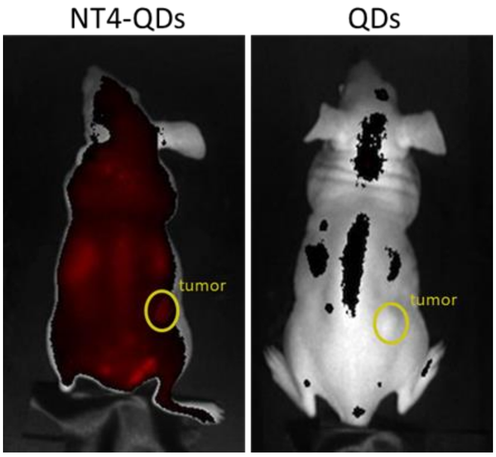

Figure S3. In vivo imaging of HT29 tumor-xenograft mice (yellow circles) at 3 h post-injection of 200 pmol of NT4-QDs (left) or free QDs (right).
